# Cytokines, Proliferation Markers, Antimicrobial Factors and Neuropeptide-Containing Innervation in Human Nasal Mucosa after Rhinoseptoplasty Procedure

**DOI:** 10.3390/medsci9020025

**Published:** 2021-04-21

**Authors:** Marija Podlesnaja, Mara Pilmane, Gunta Sumeraga

**Affiliations:** 1 Institute of Anatomy and Anthropology, Riga Stradins University, LV-1010 Riga, Latvia; pilmane@latnet.lv; 2 Department of Otorhinolaryngology, Pauls Stradiņš Clinical University Hospital, LV-1002 Riga, Latvia; Gunta.Sumeraga@stradini.lv

**Keywords:** nose, mucosa, healthy, human, cytokines, epithelium, innervation

## Abstract

The nasal cavity lined by nasal mucosa, is a significant part of respiratory system of human. However, there are no studies aimed to detect a molecular phenotype of healthy and normal functioning nasal mucosa, obtained after rhinoseptoplasty procedure, to understand its physiology and growth and inflammation processes. Thus, our aim is to identify human healthy nasal mucosa cytokines, neuropeptide-containing innervation and cell proliferation markers to form a control group for further tissue investigation of human nasal polyposis as the next step of our research. The study included surgery materials from 17 healthy humans. Biotin-streptavidin immunohistochemistry was performed for detection of tissue PGP9.5, Ki-67, β-Defensin 2, IL-1, IL-4, IL-6, IL-7, IL-8, IL-10, IL-12. Results were evaluated semi-quantitatively and by Friedman ANOVA and Spearman rang correlation tests. All factors were more widely expressed by superficial epithelium than by glandular one. Abundance of ILs-8, -10 and -12 positive cells was detected in comparison with moderate to numerous distributions of IL-1, IL-6 and β-Defensin 2. Moderate number of PGP 9.5-containing nerve fibers and only few to moderate Ki-67 positive cells were found in healthy nasal mucosa. We revealed statistically significant difference between Ki-67 and ILs-4, -6, -7, -8, -10, -12 both in healthy nasal mucosa superficial and glandular epithelium. From nasal epithelia, commonly the surface one displays more cytokines and β-Defensin 2 in comparison to the glandular one. Numerous to abundant expression of ILs-4, -6, -7, -8, -10, -12 and β-Defensin 2 in nasal superficial and glandular epithelia proves probably these factors’ role into the common immune response of tissue and stimulation of immune cell differentiation.

## 1. Introduction

The nasal cavity is a significant part of respiratory system of human. Its structures function both to allow air to enter the respiratory tract, to regulate its flow and also to make the sense of smell [1]. Nasal cavity is lined by nasal mucosa. It functions like a piece of respiratory mucosa that is mucous membrane that lines also the respiratory tract [2]. It participates in mediating immune responses to infectious particles and allergens that enter the nose. Both IgA- and IgE-containing mucus and normal bacterial flora in the nasal mucosa play an important role in the defense of respiratory tissues [3]. Finally, this mucosa is closely attached to periosteum and perichondrium of conchae nasales.

The nasal cavity’s epithelium is divided into two types, according to its functions [4]. The first type is respiratory epithelium that is ciliated pseudostratified columnar epithelium, and it serves to protect and moisten the airways [5]. The second type is olfactory epithelium that participates in transduction of olfactory information. In contrast to respiratory epithelium that lacks neurons and functions in particular as a protective surface, olfactory epithelium is formed by some distinct cell types, including the olfactory receptor neurons, basal and sustentacular cells. The internal surface of nasal cavity is lined by mucus that is produced by Bowman’s glands [6].

It is very important to detect a molecular phenotype of healthy and normal functioning nasal mucosa to understand its physiology and growth, and inflammation processes there. For example, Vlachostergios (2011) wrote that neurons and the diffuse neuroendocrine system is rich with PGP 9.5. It is a neuro-specific protein that functions to prevent ubiquitinated peptides from degradation by proteasomes by removing the ubiquitin from them. Protein abundance in nerves has been used as a marker for nerve fibers [7]. Holbrook et al., 2011 in their research on immunohistochemical characterization of human olfactory tissue described that there is a notable difference between PGP 9.5 staining in respiratory and olfactory region. Therefore, human olfactory mucosa is located in the upper part of nasal cavity below the cribriform plate and there are some areas also in this region that lack PGP 9.5 positive cells. As these PGP 9.5 negative regions are interspersed with PGP 9.5 positive areas, it was assumed that “negative” regions correspond to ducts of Bowman’s glands. While PGP 9.5 staining was restricted by neuroepithelium, still some sporadic PGP 9.5-positive epithelial cells were detected. These non-neuronal cells shew markers for respiratory epithelium [8].

The other peptides that are expressed by myeloid and epithelial cells, are human defensins. They are antibiotic peptides that have been already detected in Paneth cells, in the crypts of Lieberkühn and in the female reproductive tract [9]. The research on human alpha-defensin 5 (HD5) mRNA expression in nasal and bronchial epithelial cells showed that HD5 presence is volatile and seems to be locally induced. The authors underlined that HD5 might play an important role in innate defense mechanism in nasal epithelium [10].

Ki-67 is a nuclear protein that is associated with cell proliferation processes and, moreover, it is connected with ribosomal RNA transcription and its inactivation can lead to stop of the ribosomal RNA synthesis [11]. Mrówka-Kata et al., 2007 analyzed immunohistochemical manifestation of proliferation marker Ki-67 in healthy nasal mucosa and nasal polyps’ tissues and didn’t find a statistical considerable difference between Ki-67 positive cells in healthy and polyp’s tissue. Proliferating cells were detected mainly in the epithelium and a few in subepithelium and named a planoepithelial metaplasia [12]. On the contrary, cell cycle regulatory protein expression research in normal nasal mucosa and nasal polyps detected significant difference in proliferating epithelial cells number in polyps, in comparison with healthy tissue. In addition to an abundance of Ki-67 positive cells, an overexpression of p53, MDM2 (mouse double minute 2 homolog) and Bcl-2 (B-cell lymphoma 2 protein) was found too, what means that an increase in apoptosis also was present in polyps’ tissues [13].

Interleukins (ILs) are produced by both leukocytes and many different cells of human organism. They participate in activation, differentiation, migration, and adhesion processes of immune cells, as well as in development of inflammation and anti-inflammation processes [14]. It is necessary to reveal both pro- and anti-inflammatory interleukin distribution in explored tissue, to understand fundamental processes that take place in nasal mucosa. Pro-inflammatory interleukin 1 alpha (IL-1α) is significantly responsible for sepsis promotion [15]. It is mainly produced by macrophages, neutrophils, and epithelial cells [16]. IL-6 usually acts as both a pro-inflammatory and anti-inflammatory cytokine-through its inhibitory effects on TNF-alpha and IL-1, and activation of IL-10 [17]. IL-8 is mostly produced by macrophages, epithelial and airway smooth muscle cells [18]. Two basic functions of IL-8 include chemotaxis induction and phagocytosis stimulation [19].

Equally intriguing is interleukin 4 (IL-4) detection in human nasal mucosa. It is a cytokine which induces naïve T-helper differentiation to Th2 in a positive feedback way [20]. The production of IgE antibody is directly related to the balance between IL-4 and IFN-gamma [21].

Being similar to IL-8, IL-7 is also a hematopoietic growth factor and it is usually secreted by stromal cells in thymus and in the bone marrow, and also was detected to be produced by dendritic cells, hepatocytes and epithelial cells, but not by lymphocytes; it maintains survival of naive and memory T-cell homeostasis in the periphery and regulates the proliferation and differentiation of lymphocytes within the mucosal layers [22]. Thus, it can be used in human mucosa investigations together with IL-1 and IL-8 to detect inflammation processes. Bikker et al., 2012 described IL-7 capacity to induce TNF-alpha and accentuate IL-7 important mediator role in several chronic inflammatory diseases. [23] In the study by Vaitkus et al., 2019 on changes of inflammatory markers of the nasal mucosa in patients with different-origin sinusitis emphasized that IL-7 concentration was statistically significantly higher in chronic sinusitis group comparing to control group result [24].

Interleukin 10 (IL-10) functions as an anti-inflammatory cytokine, its expression in nasal epithelium demonstrated negative correlation with clinical symptoms in patients with house dust mite allergy, thus IL-10 probably regulates allergic symptoms, and its pathways disorders targeting treatment can reveal new ways of treatment of allergies [25]. On the contrary, being an interleukin that is naturally secreted by dendritic cells, macrophages and neutrophils, IL-12 effectively participates in modulating nasal allergic response [26].

Our aim was for this work was the identification of those human healthy nasal mucosa cytokines, neuropeptide-containing innervation factors, and cell proliferation markers what will make possible the comparison of different mucosal tissue factor’s distribution and appearance with such in the disease affected tissue. This is also the main novelty of our paper and prepares the basis for future research on nasal polyposis.

## 2. Materials and Methods

The study included surgery materials from 17 healthy humans. The tissues were obtained from nasal mucosal biopsies (contained fragments of the respiratory epithelium and subepithelium of the mucosa) taken before the rhinoseptoplasty procedure in Department of Otorhinolaryngology of Pauls Stradins Clinical University Hospital, the study was conducted at the Department of Morphology of Riga Stradins University, Latvia.

This study was independently reviewed and approved by the local Ethical Committee of the Riga Stradins University 2010, No. E-9 (2); 2 September 2010. All of the patients gave their informed consent to participate in the study after the nature of the study had been fully explained.

Tissues were fixed for a day in a mixture of 2% formaldehyde and 0.2 picric acid in 0.1 M phosphate buffer (pH 7.2). Afterwards, they were rinsed in thyroid buffer, containing 10% saccharose for 12 h and then embed into paraffin. Three micrometer thin sections were cut, which were then stained with hematoxylin and eosin (H&E) for morphological evaluation. Biotin-Streptavidin biochemical method was used for immunohistochemistry (IMH) to detect: PGP 9.5 (439273A, 1:200, Invitrogen, Carlsbad, CA, USA), Ki-67 (1325506A, 1:100, Cell Marque, Rocklin, CA, USA), β-Defensin 2 (sc-20798, 1:100, Santa Cruz Biotechnology Inc., Dallas, TX, USA), IL-1 (orb308737, 1:100, Biorbyt, Cambridge, UK), IL-4 (orb10908, 1:100, Biorbyt, Cambridge, UK), IL-6 (sc-130326, 1:100, Santa Cruz Biotechnology Inc., Dallas, TX, USA), IL-7 (orb13506, 1:100, Biorbyt, Cambridge UK), IL-8 (orb39299, 1:100, Biorbyt, Cambridge UK), IL-10 (250713, 1:100, BioSite, Solihull, UK), IL-12 (orb10894, 1:100, Biorbyt, Cambridge UK) [27]. The slides were analyzed by light microscopy. The results were evaluated by grading the appearance of positively stained cells in the visual field [28]. No positive structures in the visual field were labelled as 0, rare positive structures were labelled with 0/+, few positive structures: +, few to moderate number of positive structures in the visual field: +/++, moderate number of positive structures in the visual field: ++, moderate to numerous positive structures in the visual field: ++/+++, numerous positive cells in the visual field: +++, numerous number to abundance of structures in the visual field: +++/++++ and abundance of positive structures in the visual field was labelled as ++++. Histological variations in nasal epithelium were determined semi-quantitatively by using the grading system of Peebua et al., 2006, which was slightly modified [29].

The data processing was performed with SPSS software, version 22.0 (IBM Company, Chicago, IL, USA). Spearman’s rank correlation coefficient was used to determine correlations between factors, where *r* = 0–0.2 was assumed as a very weak correlation, *r* = 0.2–0.4—a weak correlation, *r* = 0.4–0.6—a strong correlation and *r* > 0.6—a very strong correlation. Friedman ANOVA was used to reveal statistically significant differences between different factors. The level of significance for tests was chosen as 5% and 1% (*p*-value < 0.05 and <0.01).

## 3. Results

### 3.1. Routine Changes

**Figure 1:**
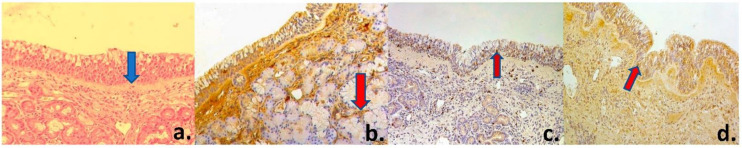
Human healthy nasal mucosa with unchanged basal membrane (arrow) and without any inflammation in routine staining (H&E, ×200) (**a**), with moderate number of PGP 9.5-containing nerve fibers (arrow) (PGP 9.5 IMH, ×200) (**b**), with few to moderate number of Ki-67 positive cells (arrow) in the superficial epithelium (Ki-67 IMH, ×200) (**c**), and numerous number of β-Defensin positive superficial epithelial cells (arrow) (β-Defensin 2 IMH, ×200) (**d**).

Human healthy nasal mucosa showed mainly epithelial and subepithelial connective tissue without any inflammation signs or other abnormalities (Figure 1a).

### 3.2. Immunohistochemical Changes. Innervation and Proliferation

A moderate (++) number of PGP 9.5-containing nerve fibers was found in healthy nasal mucosa, though there were some variations with few (+) to numerous (+++) number of PGP 9.5 positive nerve fibers (Figure 1b) (Table 1). Nasal cavity mucosa contained few to moderate (+/++) number of Ki-67 positive cells in the superficial epithelium (Figure 1c), while there were rare (0/+) Ki-67 positive structures in glandular epithelium. This observation reveals to us the intensity of proliferation processes in the human nasal mucosa.

### 3.3. Immunohistochemical Changes. Antimicrobial Factors and Interleukins

**Figure 2:**
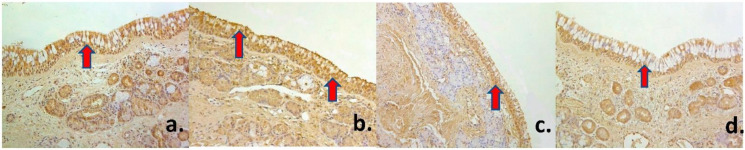
Healthy nasal mucosa presenting numerous number of IL-1 positive superficial cells (arrow) (IL-1 IMH, ×200) (**a**), abundancy of IL-4 positive cells (arrows) in superficial epithelium (IL-4 IMH, ×200) (**b**), numerous number of IL-6 positive cells (arrow) in superficial epithelium (IL-6 IMH, ×200) (**c**), and numerous number of IL-7 positive epithelial cells (arrow) in healthy nasal mucosa. (IL-7 IMH, ×200) (**d**).

**Figure 3:**
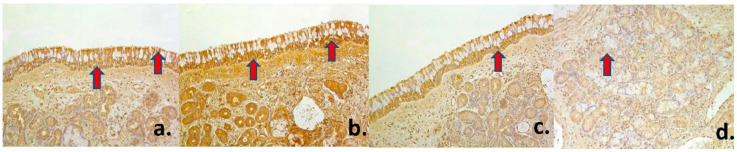
Healthy human nasal mucosa showing numerous numbers to abundancy of IL-8 positive cells (arrows) (IL-8 IMH, ×200) (**a**), abundancy of IL-10 positive cells (arrows) (IL-10 IMH, ×200) (**b**)**,** abundancy of IL-12 positive cells (arrow) superficial epithelium (IL-12 IMH, ×200) (**c**), and abundancy of IL-12 weakly stained positive cells (arrow) in glandular epithelium. (IL-12 IMH, ×200) (**d**).

A numerous (+++) number of both antimicrobial β-Defensin 2 and proinflammatory IL-1 positive superficial epithelial cells was found in healthy mucosa (Figure 1d, Figure 2a). However, glandular epithelium showed only few to moderate (+/++) β-Defensin 2 positive cells, while there was moderate to numerous (++/+++) number of IL-1 positive cells. An abundance (++++) of IL-4 positive cells was found in superficial mucosal epithelium, but only moderate (++) number was detected in the glandular one (Figure 2b). There was a numerous (+++) number of both pro- and anti-inflammatory IL-6 positive cells in the superficial epithelium, while glandular epithelium showed moderate (++) number of such cells (Figure 2c). Similar distribution was described according to IL-7 and IL-8, which showed numerous to abundance (+++/++++) of IL-8 positive cells in healthy nasal mucosa superficial epithelium that reveals us tissue tendency to become inflamed (Figure 2d, Figure 3a). In turn, both anti-inflammatory IL-10 and pro-inflammatory IL-12 factors were widely expressed in superficial nasal mucosa and were detected in abundance (++++) of cells, though only moderate (++) number of glandular epithelium cells was IL-10 and IL-12 positive (Figure 3b–d) (Table 1).

### 3.4. Statistics

A statistically significant difference was found in healthy nasal mucosa superficial epithelium between Ki-67 and ILs 4, 6, 7, 8, 10, 12 (*p*-value < 0.05); PGP 9.5 and ILs 7, 8, 10 (*p*-value < 0.05), IL-1 and IL-10 (*p*-value < 0.05). A similar statistically significant difference was detected in healthy nasal mucosa glandular epithelium, between Ki-67 and ILs 1, 4, 6, 7, 8, 10, 12 (*p*-value < 0.05), β-Defensin and IL-8, IL-10 (*p*-value < 0.05).

### 3.5. Correlations

A strong correlation was detected between PGP 9.5 and β-Defensin 2, IL-10; between Ki-67 and IL-12; between β-Defensin 2 and IL-1 in superficial epithelium. It was also found between following interleukins: IL-1 and IL-6, IL-8, IL-12; IL-4 and IL-6; IL-6 and IL-8, IL-10; IL-8 and IL-12. In glandular epithelium, a strong correlation was found between Ki-67 and IL-4; β-Defensin 2 and IL-8. A very strong positive correlation was found in healthy nasal mucosa superficial epithelium between PGP 9.5 and IL-12, IL-8 and IL-10; while in glandular epithelium it was only between IL-1 and IL-8 factors (Table 2).

## 4. Discussion

The rhinoplasty procedure is one of the most popular surgical procedures all over the world. For example, more than 200 thousand surgeries were performed in the US only in 2018 [30]. The main motivation for passing the rhinoseptoplasty procedure has always been a great variety of macroscopic anatomical disorders, such as septum deviation or a deviated nose, postinfectious nose deformations, a saddle or a flat nose etc. [31]. These and other macroscopic nose deviations’ reparation in patients, that have no clinical picture of any rhinitis or other otorhinolaryngological conditions, gives us the possibility to obtain human healthy nasal mucosa material.

Interleukins (ILs) participate in activation and correct functioning of immune cells, as well as in development of inflammation and anti-inflammation processes [14]. According to our results, superficial epithelium of healthy nasal mucosa displayed abundant expression of ILs-4, -10, -12, in comparison with glandular epithelium. Suggesting the different roles of that two types of epithelium as the main function of superficial epithelium is protection against mechanical, chemical, and microbial threat, an abundance of these pro- and anti-inflammatory ILs displays their promotion in correct and well-balanced superficial epithelium defensive reactions [32].

According to Bradding et al., 1993 who researched immunolocalization of cytokines in the nasal mucosa of normal and perennial rhinitic subjects, the immunohistochemical staining showed numerous cells immunoreactive for IL-4 and IL-8, both in rhinitic biopsies and healthy nasal mucosa materials [33]. Shen et al. (2010) found that the levels of IL-4 in allergic rhinitis and nasal septal deviation groups were significantly higher than the healthy controls [34]. In turn, Wright et al., 1999 detected that IL-12 plays a role in the in vivo suppression of the allergic inflammatory response in nasal mucosa epitheliums [35]. These studies clearly demonstrate pro-inflammatory action of IL-4 and its role in pathogenesis of many nasal disorders, as well as anti-inflammatory peculiarity of IL-12 that is both pro- and anti-inflammatory cytokine.

We did not ascertain any inflammation processes in the tissues, and a clinical picture of all patients of the study proved them to be healthy, that also confirmed the effectiveness of their nasal epithelium defensive reactions. However, we must state that numerous number to abundancy of IL-7 and -8 positive cells could proves nasal mucosa tendency to be inflamed. A very strong positive correlation was found between ILs-8 and -10 (*p* = 0.001), which proves these pro- and anti-inflammatory cytokines balance in healthy nasal mucosa. This is suspected also by Howell and Rose-Zerilli (2006), who have described the same correlations between ILs-8 and -10 in another human mucosa [36]. Interestingly, Xu et al., 2016 accentuate an increased expression of IL-10 and IL-10 related B cell activation to indicate that IL-10, a potent anti-inflammatory cytokine, plays a crucial role in the pathogenesis of some pathological processes, for example, chronic rhinosinusitis with nasal polyps [37]. We can find the similar idea in Wang et al., 2018 study, which concluded impaired IL-10 production by M2 macrophages may contribute to sustained inflammation in eosinophilic chronic rhinosinusitis with nasal polyps [38]. The abundance of both pro-inflammatory IL-12 and IL-1, anti-inflammatory IL-10 factor let us speculate on the balance between inflammatory and anti-inflammatory processes in the tissues of the healthy nasal mucosa, which can serve as the baseline expression of these factors in a control group.

Kohlgraf et al., 2010 indicated, that defensins first bind to microbial antigens attenuating their inflammation-inducing capacities, in this way by altering the connection between antigen and epithelial cells and reduce the production of proinflammatory cytokines [39]. In addition, we speculate on similar function due to the observed strong positive correlation between proinflammatory ILs-1 and -8 and β-Defensin 2 both in superficial and glandular epithelia, where β-Defensin 2 seems to show a tendency to attenuate proinflammatory cytokine responses. Guani-Guerra et al., 2011 explored β-Defensin 2 concentration in nasal lavage fluids correlation to bacterial lysates levels and detected β-Defensin 2 to be predominantly localized to the superficial epithelium [40]. In our study, we also found numerous numbers of β-Defensin 2 positive superficial epithelial cells.

Chen et al., 2020 described an interesting correlation between abundance of PGP 9.5-containing nerves in olfactory epithelium and high level of ACE-2 expression and, in turn, very low density of PGP 9.5-positive structures in respiratory epithelium, as it was also found in our study, and barely detection of ACE-2 in respiratory epithelium [41].

Healthy nasal epithelium showed only moderate PGP 9.5-containing nerves in our patients, that strongly correlated with β-Defensin 2 and very strongly with IL-12 levels in superficial epithelium. It could be accepted as a basic picture of healthy nasal tissue demonstrating indistinct neuropeptides-containing innervation and distinct antimicrobial peptide expression. However, PGP 9.5 containing innervation can be modulated by inflammation and, possibly, could increase with inflammation process development [42]. Therefore, we can assume that inflammation process can incite PGP 9.5-containing innervation development and also increase ACE-2 expression that, for its part, can make human more vulnerable for SARS-CoV-2 infection and COVID-19-associated anosmia.

De Gabory et al., 2008 found the most significant population of Ki-67 positive cells to be disseminated in the superficial epithelium [43]. An indistinctly low intensity of nasal epithelial cell proliferation was detected in our study and there were no significant correlations between Ki-67 and other tissue factors. Interestingly, there was detected a perceptible difference in number of Ki-67 positive cells in healthy nasal mucosa, comparing to nasal polyps and nasal metaplasia [13]. Oncel et al., 2011 in their research on evaluation of P53, P21 and Ki-67 factors paranasal sinus squamous cell carcinoma and inverted papilloma proved also that Ki-67 proliferative index values were significantly higher in the squamous cell carcinoma compared with non-malignant processes [44]. Thus, we explain the presence of moderate number of Ki-67 positive cells in healthy nasal mucosa as a basic indicator for malignation.

Many studies concerning the morphological properties of pathologies of the human nasal mucosa require a control group, which is a morphological picture of a healthy mucosa, including markers of inflammation, innervation, and proliferation. The novelty of our work is the detection of general picture in distribution and appearance pro- and anti-inflammatory interleukins, a marker of proliferation (Ki-67) and innervation (PGP9.5), to make such a comparison available for the various pathologies. At the next stage of our study, we plan to compare the immunohistochemical picture and correlations between the factors of primary and recurrent polyps of the nasal mucosa with the results we obtained from the control group of healthy nasal mucosa.

Our study also showed several limitations. For example, there is a great variety of environmental factors which correlation to the appearance of different tissue factors in healthy nasal mucosa stay still unclear. Secondly, there is a significant lack of prior re-search studies on the healthy human nasal mucosa what could be explained by the strict ethical issues on the obtainment of human material based only on voluntary principle and time-consuming process. Thirdly, the semi-quantitative method of analysis also might be considered partially subjective. However, this was the most suitable way to determinate histological tissue factor patterning as some other preferable method (for instance, the factor concentration detection by ELISA) requests the obtainment of much larger tissue samples what is not acceptable from the point of view of ethical/patient interests.

## 5. Conclusions

The rich baseline expression of IL-4, IL-6, IL-7, and especially IL-8, IL-10 and IL-12 in superficial epithelium overseeds the expression of these cytokines in the glandular epithelium of healthy nasal mucosa, suggesting the involvement of these cytokines into the local immune response and stimulation of immune cell differentiation.

Abundance expression of main anti-inflammatory cytokine IL-10 together with numerous numbers of antimicrobial peptide β-Defensin 2 positive cells over the moderate to numerous numbers of pro-inflammatory cytokines proves the baseline dominance of anti-inflammatory suppression in the healthy nasal mucosa.

Healthy nasal mucosa possesses scarce neuropeptide-containing innervation and indistinct proliferation of epithelial cells.

## Figures and Tables

**Table 1:** Relative number of cytokines, proliferation markers, antimicrobial factors, and neuropeptide-containing innervation in human healthy nasal mucosa.

	Factors	PGP 9.5	Ki-67		β-Defensin 2		IL-1		IL-4		IL-6		IL-7		IL-8		IL-10		IL-12	
Subject №																				
Type of Tissue Nasal Mucosa		Nasal Mucosa	Superficial Epithelium	Glandular Epithelium	Superficial Epithelium	Glandular Epithelium	Superficial Epithelium	Glandular Epithelium	Superficial Epithelium	Glandular Epithelium	Superficial Epithelium	Glandular Epithelium	Superficial Epithelium	Glandular Epithelium	Superficial Epithelium	Glandular Epithelium	Superficial Epithelium	Glandular Epithelium	Superficial Epithelium	Glandular Epithelium
1		++	++/+++	0	+++	+/++	+++	+	+++	++	+++	+	++	++	++	++	+++	+++	++++	++
2		+++	+	+	+++	+	+++	+/++	+++	+++	++++	+/++	+++	+/++	++++	++	++++	++	+++	+/++
3		++	+	0/+	+++	+	++	+	++++	++	++/+++	+	++++	++	+++	++	++++	+/++	+++	++
4		++	+	0	+++	+	+++	+	+++	++	++++	++	++++	++	++++	++	++++	+++	+++	++
5		+++	++	0	+++	+	++	+	+++	++	++	+	+++	++	+++	++	+++	+++	++	++
6		+	0/+	0/+	++++	+	+++	++	+++	+/++	++++	+/++	++	++	+++	++	++++	+	+++	+
7		++	+/++	+	+++	++	++	+++	+++	++	+++	++	+++	+/++	+++	+++	++++	++	++	+
8		+++	+/++	+	++	+	+	++	++++	+++	++	++	+++	++/+++	++	++	+++	++	++	+
9		+++	+	0	++	+	+++	+++	+++	++	+++	+	++++	++	+++	++	+++	++	+++	+/++
10		+++	++	0	++++	++	+++	+++	+++	++	++++	++/+++	++++	++/+++	++++	+++	++++	++++	+++	+
11		++	+	0	+++	+	+++	+++	+++	++	++++	+++	+++	++	++++	+++	++++	++++	+++	++/+++
12		+	++	0	++++	+++	+++	+++	++++	++	+++	+	++++	+++	++++	+++	++++	+++	++++	++
13		+	++/+++	0/+	+++	+	+++	++	+++	+++	++++	++	+++	++	++++	+++	++++	+++	++++	+++
14		++	++/+++	0/+	+++	++	+++	++	++++	++	+++	++	++++	++	++++	++	++++	++	++++	++
15		+	+++	+	++++	++	+++	+++	++++	++	+++	++	+++	++	++++	+++	++++	+++	++++	+++
16		++	+	+	++	+	++	++	+++	+++	++++	++	++++	++	++++	++	++++	++++	++++	++
17		+/++	++	0	+++	+	+++	+++	+++	++	+++	++	++++	+++	++++	++	++++	+++	++++	+++
Average		++	+/++	0/+	+++	+/++	+++	++/+++	++++	++	+++	++	+++	++	+++/++++	++	++++	+++	++++	++

Abbreviations: IL—interleukin; PGP 9.5—protein gene product 9.5; 0—no positive structures in the visual field; 0/+—rare positive structures; +—few positive structures; +/++—few to moderate number of positive structures in the visual field; ++—moderate number of positive structures; ++/+++—moderate to numerous positive structures; +++—numerous positive cells; +++/++++—numerous number to abundance of structures; ++++—abundance of positive structures in the visual field.

**Table 2:** Spearman’s rank correlation coefficient between the relative numbers of different tissue factors in healthy human mucosa.

Factor 1	Factor 2	R	*p-* Value
Strong positive correlation in superficial epithelium			
PGP 9.5	β-Defensin	0.506	0.038
PGP 9.5	IL-10	0.539	0.026
Ki-67	IL-12	0.486	0.048
β-Defensin 2	IL-1	0.492	0.045
IL-1	IL-6	0.538	0.026
IL-1	IL-8	0.513	0.035
IL-1	IL-12	0.535	0.027
IL-4	IL-6	0.554	0.021
IL-6	IL-8	0.596	0.012
IL-6	IL-10	0.579	0.015
IL-8	IL-12	0.518	0.033
Strong positive correlation in glandular epithelium			
Ki-67	IL-4	0.484	0.049
β-Defensin 2	IL-8	0.549	0.023
A very strong positive correlation in superficial epithelium			
PGP 9.5	IL-12	0.628	0.007
IL-8	IL-10	0.741	0.001
A very strong positive correlation in glandular epithelium			
IL-1	IL-8	0.638	0.006

Abbreviations: IL—interleukin; PGP 9.5—protein gene product 9.5.

## References

[1] Klocke R.A., Siebens A.A., Heath D.A., Beers M.F., Burri P.H., Elliott D.H., Weibel E.R., Cherniack N.S. Human respiratory system. Encyclopedia Britannica.

[2] Beule A.G. (2011). Physiology and pathophysiology of respiratory mucosa of the nose and the paranasal sinuses. GMS Curr. Top. Otorhinolaryngol. Head Neck Surg..

[3] Freeman S.C., Karp D.A., Kahwaji C.I. (2008). Nasal Physiology.

[4] Scherzad A., Hagen R., Hackenberg S. (2019). Current Understanding of Nasal Epithelial Cell Mis-Differentiation. J. Inflamm. Res..

[5] Mescher A.L. (2018). The Respiratory System. Junqueira’s Basic Histology: Text & Atlas.

[6] Purves D., Augustine G.J., Fitzpatrick D. (2001). The Olfactory Epithelium and Olfactory Receptor Neurons. Neuroscience.

[7] Vlachostergios P.J., Papandreou C.N. (2011). Neuroendocrine differentiation and the ubiquitin-proteasome system in cancer: Partners or enemies?. World J. Exp. Med..

[8] Holbrook E.H., Wu E., Curry W.T., Lin D.T., Schwob J.E. (2011). Immunohistochemical characterization of human olfactory tissue. Laryngoscope.

[9] Ouellette A.J. (2011). Paneth cell α-defensins in enteric innate immunity. Cell. Mol. Life Sci..

[10] Freye M., Bargon J., Dauletbaev N., Weber A., Wagner T.O., Gropp R. (2000). Expression of human alpha-defensin 5 (HD5) mRNA in nasal and bronchial epithelial cells. J. Clin. Pathol..

[11] Rahmanzadeh R., Hüttmann G., Gerdes J., Scholzen T. (2007). Chromophore-assisted light inactivation of pKi-67 leads to inhibition of ribosomal RNA synthesis. Cell Prolif..

[12] Mrówka-Kata K., Namysłowski G., Stęplewska K., Gabriel A., Wysocka A. (2007). Immunohistochemiczna ocena moleku?y Ki67 w tkance polipów nosa. Otolaryngol. Polska.

[13] Garavello W., Viganò P., Romagnoli M., Sordo L., Berti E., Tredici G., Gaini R.M. (2005). Expression of Cell Cycle Regulatory Proteins and Analysis of Apoptosis in Normal Nasal Mucosa and in Nasal Polyps. Am. J. Rhinol..

[14] Justiz Vaillant A.A., Qurie A. (2019). Interleukin.

[15] Dinarello C.A. (2018). Overview of the IL-1 family in innate inflammation and acquired immunity. Immunol. Rev..

[16] Di Paolo N.C., Shayakhmetov N.C.D.P.D.M. (2016). Interleukin 1α and the inflammatory process. Nat. Immunol..

[17] Muñoz-Cánoves P., Scheele C., Pedersen B.K., Serrano A.L. (2013). Interleukin-6 myokine signaling in skeletal muscle: A double-edged sword?. FEBS J..

[18] Liu C., Zhang X., Xiang Y., Qu X., Liu H., Liu C., Tan M., Jiang J., Qin X. (2018). Role of epithelial chemokines in the pathogenesis of airway inflammation in asthma (Review). Mol. Med. Rep..

[19] Beste M.T., Lomakina E.B., Hammer D.A., Waugh R.E. (2015). Immobilized IL-8 Triggers Phagocytosis and Dynamic Changes in Membrane Microtopology in Human Neutrophils. Ann. Biomed. Eng..

[20] Sokol C.L., Barton G.M., Farr A.G., Medzhitov R. (2007). A mechanism for the initiation of allergen-induced T helper type 2 responses. Nat. Immunol..

[21] Mitsias D.I., Tzioufas A.G., Veiopoulou C., Zintzaras E., Tassios I.K., Kogopoulou O., Moutsopoulos H.M., Thyphronitis G. (2002). The Th1/Th2 cytokine balance changes with the progress of the immunopathological lesion of Sjogren’s syndrome. Clin. Exp. Immunol..

[22] Alves N.L., Goff O.R.-L., Huntington N.D., Sousa A.P., Ribeiro V.S.G., Bordack A., Vives F.L., Peduto L., Chidgey A., Cumano A. (2009). Characterization of the thymic IL-7 niche in vivo. Proc. Natl. Acad. Sci. USA.

[23] Bikker A., Hack C.E., Lafeber F.P., Van Roon J.A. (2012). Interleukin-7: A key Mediator in T Cell-driven Autoimmunity, Inflammation, and Tissue Destruction. Curr. Pharm. Des..

[24] Vaitkus J., Vaitkus S., Vitkauskienė A. Changes of inflammatory markers IL-4, IL-5, IL-7, IL-21 of the nasal mucosa in patients with chronic odontogenic and fungal-allergic sinusitis. Proceedings of the 5th Lithuanian—Polish ENT Congress.

[25] Muller B., De Groot E.J.J., Kortekaas I.J.M., Fokkens W.J., Van Drunen C.M. (2007). Nasal epithelial cells express IL-10 at levels that negatively correlate with clinical symptoms in patients with house dust mite allergy. Allergy.

[26] Hamza T., Barnett J.B., Li B. (2010). Interleukin 12 a Key Immunoregulatory Cytokine in Infection Applications. Int. J. Mol. Sci..

[27] Guesdon J.L., Ternynck T., Avrameas S. (1979). The use of avidin-biotin interaction in immunoenzymatic techniques. J. Histochem. Cytochem..

[28] Pilmane M., Rumba I., Sundler F., Luts A. (1998). Patterns of distribution and occurrence of neuroendocrine elements in lungs of hu-mans with chronic lung diseases. Proc. Latv. Acad. Sci..

[29] Peebua P., Kruatrachue M., Pokethitiyook P., Kosiyachinda P. (2006). Histological effects of contaminated sediments in Mae Klong River Tributaries, Thailand, on Nile tilapia, Oreochromis niloticus. Sci. Asia.

[30] Fichman M., Piedra Buena I.T. (2020). Rhinoplasty.

[31] Hong H.R., Kim S.H., Kim J.H., Jang Y.J. (2015). Aesthetic Motivation of Geriatric Rhinoplasty the Surgical Outcome. J. Craniofacial Surg..

[32] Standring S. (2016). Gray’s Anatomy: The Anatomical Basis of Clinical Practice.

[33] Bradding P., Feather I.H., Wilson S., Bardin P.G., Heusser C.H., Holgate S.T., Howarth P.H. (1993). Immunolocalization of cytokines in the nasal mucosa of normal and perennial rhinitic subjects. The mast cell as a source of IL-4, IL-5, and IL-6 in human allergic mucosal inflammation. J. Immunol..

[34] Shen J., Hong S. (2010). Serum levels of IL-12, IL-4 and pathologic changes by scanning electron microscope of nasal mucous inflammation. Lin Chuang Er Bi Yan Hou Tou Jing Wai Ke Za Zhi.

[35] Wright E.D., Christodoulopoulos P., Frenkiel S., Hamid Q. (1999). Expression of interleukin (IL)-12 (p40) and IL-12 (beta 2) receptors in allergic rhinitis and chronic sinusitis. Clin. Exp. Allergy.

[36] Howell W.M., Rose-Zerilli M.J. (2006). Interleukin-10 polymorphisms, cancer susceptibility and prognosis. Fam. Cancer.

[37] Xu J., Han R., Kim D.W., Mo J.-H., Jin Y., Rha K.-S., Kim Y.M. (2016). Role of Interleukin-10 on Nasal Polypogenesis in Patients with Chronic Rhinosinusitis with Nasal Polyps. PLoS ONE.

[38] Wang Z.-C., Yao Y., Wang N., Liu J.-X., Ma J., Chen C.-L., Deng Y.-K., Wang M.-C., Liu Y., Zhang X.-H. (2018). Deficiency in interleukin-10 production by M2 macrophages in eosinophilic chronic rhinosinusitis with nasal polyps. Int. Forum Allergy Rhinol..

[39] Kohlgraf K.G., Pingel L.C., Dietrich D.E., Brogden K.A. (2010). Defensins as anti-inflammatory compounds and mucosal adjuvants. Future Microbiol..

[40] Guaní-Guerra E., Negrete-García M.C., Montes-Vizuet R., Asbun-Bojalil J., Terán L.M. (2011). Human β-Defensin-2 Induction in Nasal Mucosa after Administration of Bacterial Lysates. Arch. Med Res..

[41] Chen M., Shen W., Rowan N.R., Kulaga H., Hillel A., Jr M.R., Lane A.P. (2020). Elevated ACE2 expression in the olfactory neuroepithelium: Implications for anosmia and upper respiratory SARS-CoV-2 entry and replication. Eur. Respir. J..

[42] Moynes D.M., Lucas G.H., Beyak M.J., Lomax A.E. (2013). Effects of Inflammation on the Innervation of the Colon. Toxicol. Pathol..

[43] De Gabory L., Deminière C., Stoll D. (2008). Expression immunohistochimique de l’ACE, de l’UEA-I et du Ki-67 dans les papillomes inversés naso-sinusiens Immunohistochemistry expression of 3 markers (CEA, UEA-I and Ki-67) in nasal inverted papillo-mas. Rev. Laryngol. Otol. Rhinol..

[44] Oncel S., Cosgul T., Calli A., Calli C., Pinar E. (2011). Evaluation of P53, P63, P21, P27, Ki-67 in Paranasal Sinus Squamous Cell Carcinoma and Inverted Papilloma. Indian J. Otolaryngol. Head Neck Surg..

